# Predictors of fertility awareness among selected married women of childbearing age in Nigeria: a cross-sectional survey

**DOI:** 10.4314/ahs.v23i3.11

**Published:** 2023-09

**Authors:** Adaobi Uchenna Mosanya, Deborah Oyine Aluh, Chibueze Anosike, Maureen Ogochukwu Akunne, Chigozie Gloria Anene-Okeke, Abdulmuminu Isah

**Affiliations:** Department of Clinical Pharmacy and Pharmacy Management, Faculty of Pharmaceutical Sciences, University of Nigeria, Nsukka, Nigeria. PMB 410001, Enugu State, Nigeria

**Keywords:** Nigeria, fertility, educational status, menstrual cycle, internet

## Abstract

**Background:**

Increased fertility awareness can help infertile couples to achieve pregnancy.

**Objectives:**

This study aimed to determine both the predictors and levels of fertility awareness among married Nigerian women of childbearing age.

**Methods:**

A nationwide cross-sectional survey. Data were collected via online and face to face questionnaires. Descriptive and inferential analysis were done with SPSS 25.

**Results:**

Most respondents married between ages 24-29 years old (40%) and just over half had good fertility awareness (53%). The associated factors were age at menarche (X^2^ = 9.962, p = 0.007), geopolitical zone of residence (X^2^ = 17.301, p = 0.008), level of education (X^2^ = 64.843, p < 0.001), employment status (X^2^ = 9.319 p = 0.025) menstrual cycle charting (X^2^ = 66.392, p < 0.001), use of internet to increase awareness (X^2^ = 39.849, p < 0.001) and books (X^2^ = 58.855, p < 0.001). Fertility awareness was lower for those with secondary education than postgraduates (AOR=0.213, 95% CI 0.116-0.390, p < 0.001). Moreover, the odds of having good fertility awareness were less in those who did not chart their menstrual cycle (AOR=0.363, 95% CI 0.245-0.538, p < 0.001).

**Conclusion:**

Menstrual cycle charting and level of education were predictors of fertility awareness.

## Introduction

Fertility health literacy is the skill which enables individuals to acquire, understand and utilize fertility health related information for their complete well-being.^1^ Fertility health literacy is important because it facilitates the understanding of various signs and symptoms of fertile window of the menstrual cycle i.e., when sexual intercourse is most likely to result in pregnancy. This is particularly so for infertile and sub-fertile women. They can use the status of the fertile mucus to identify their peak fertile days, thereby maximizing their chances of conception. Lifestyle behaviors and education have been identified as preventable causes of infertility 2 and women usually do not seek knowledge about fertility until they marry^3^. It has been observed that women who learn how to observe and track their menstrual symptoms, are more confident in knowing whether ovulation has occurred and the general state of their fertility health^4^.

Ovulation is an important event necessary for fertility with its characteristic observable sign called the fertile cervical mucus^5^. This mucus type required for sperm survival and maturation is moist, stretchy, and clear allowing the smooth passage of the spermatozoa through the cervix to the fallopian tube. However, in its absence, the vagina is dry and acidic, creating a hostile environment for the sperm 6. In the fertile window, the lifespan of the ovum is 24-36hours while the duration of sperm's survival and ability to fertilize the ovum is about 6 days^7^. Abnormalities in ovulation followed by irregular cycles or amenorrhea are leading signs of a woman's ill health^8^. Observation of fertility signs, such as a change in the quality and quantity of the fertile cervical mucus serve as fertility indicators because they coincide with levels of reproductive hormones^9^. For example, a systematic review by Thijssen et al. reported that a study of ovulation detection in 346 women resulted in a cumulative probability of conception of 81% after 6 months and 92% after 12 months^10^.

Fertility awareness charts have been used to identify women who have reduced or the absence of cervical mucus secretion and have been treated with mucus enhancers such as Vit B6 or Guaifenesin^11^. Such charts are also used to ascertain the peak progesterone levels necessary for pregnancies to reach their full term^12^. Some international studies related to fertility awareness have been carried out amongst women seeking fertility assistance in Australia^13^, on women from the student population in Iraq, Sweden and America^14^-^17^ and those attending an immunization clinic at a tertiary teaching hospital in the Northern Nigeria^18^. Although WHO estimated that 93% of women worldwide can identify their fertile days^19^, there are no published data in Nigeria of married women's awareness and the relevance of the fertile and infertile days in their cycle.

The present study was therefore carried out among married women of childbearing age to determine the extent of fertility awareness and the potential factors which might serve as facilitators or barriers. The survey was conducted through community pharmacies because they are considered as one of the major private sector providers of contraceptive form of family planning in Nigeria^20^-^23^. In addition, efforts are been made in different sectors to second the recommendations of WHO^24^ to expand the family planning services of community pharmacists in Nigeria 25,26. It was hoped that the research might point to existing knowledge gaps regarding fertility awareness which could be filled with appropriate educational interventions by community pharmacists.

## Methods

### Study design

This study utilized a cross-sectional survey method. It was a nationwide pharmacist-led pilot study conducted among married women of childbearing age living in Nigeria. Ethical approval was obtained from the Research Ethics Committee of the Faculty of Pharmaceutical Sciences, University of Nigeria. The approval reference number was FPSRE/UNN/20/0007. No respondents' identifying information was collected during the survey. The consent of the respondents was received before data collection.

### Study setting

The study was conducted across Nigeria and inclusive of all 36 states and the Federal Capital Territory that are grouped into six geo-political zones: North-West, NorthEast, North-Central, South-West, South-East, South-South.

### Participants

The eligibility criteria were married women of childbearing age who consented to participate in the survey. As statistics about the size or demographic composition of the target population were not available nor was there any database for the target population, probability sampling methods could not be used.

### Sample size and sampling technique

The minimum sample size estimation was calculated using the formula:

S= Z^2^ p (1-p)/m^2^

S= the minimum sample size for unknown population.

Z= Z score (this was determined based on 95% confidence level) 1.96.

p = percentage of population (assumed to be 50% =0.5)

m = margin of error, which was 5%=0.05

The minimum sample size was therefore 384.

A non-probability convenience sampling technique was used to get responses across the 36 states and the Federal Capital Territory with a calculated quota size for each state of approximately 11 responses. This was arrived at by dividing the minimum sample size of 384 by 37.

### Data collection

After an extensive literature search^13^,^18^, 10 items were generated for the questionnaire to assess fertility awareness and socio-demographics. The items used to assess the fertility awareness were awareness of the fertile days in the menstrual cycle, awareness of ovulation pain, awareness of your cervical mucus secretions, awareness of the cervical mucus type which indicates fertility, awareness of ovulation detection through the urine, active attempt to increase fertility awareness through different sources of information, awareness that doctors can educate them on fertility, awareness that they can discuss with their doctors about fertility, awareness of intercourse timing to increase chances of pregnancy and awareness that fertility days can be learnt. The questionnaire was face and content-validated independently by three experts in the field. Corrections were made and copies were sent to community pharmacies in different parts of the country. An online questionnaire was also sent to participants via WhatsApp especially those who reside in the Northwest and Northeast zones. These were in areas of high insecurity due to insurgencies and terrorist attacks. Data collection lasted from June to December 2020.

### Data Analysis

At the end of collection, the data were checked for appropriateness on Microsoft Excel, coded and analysed using the Statistical Package for Social Sciences (SPSS version 25). Descriptive statistics (frequencies, percentages, measures of central tendency and dispersion) were used to summarize the sociodemographic characteristics and the awareness scores of the respondents. The Pearson Chi-square test was used to determine the relationship between the socio-demographics and fertility awareness. Univariate and multivariate logistic regression analysis was performed to determine the predictors of fertility awareness. P values less than 0.05 were considered statistically significant. The reliability test was done on the awareness questions. A Cronbach's alpha value of 0.74 was obtained.

### Study Variables

**Fertility awareness:** Correct answers for the knowledge items were given a score of ‘1”, while an incorrect answer was assigned ‘0’. The total score for each respondent was computed and descriptive analysis run to determine the measures of central tendency and dispersion. The scores were not normally distributed, and the median score was therefore used as the cut off to categorize the patients into those with good or poor fertility awareness. Those who have good fertility awareness are those who scored from the median score and above while those who had poor fertility awareness are those who scored below the median score.

**Predictors of fertility awareness:** To identify any associations between the sociodemographic data and good fertility awareness among the participants, Chi-square statistical analysis was used. All the associating factors that were significant at *p* ≤ 0.05 in the Chi-square analysis were considered for further analysis using the binary logistic regression model to determine the predictors of good fertility awareness. All the covariates that were significant at *p* < 0.25 in the bivariate analysis were considered for further multivariate analysis. A multivariable logistic regression was used to identify the independent variables that were associated with good fertility awareness. The level of statistical significance was *p* ≤ 0.05.

**Menstrual charting ability:** By this we mean that the woman charts her menstrual cycle. Only two response options were provided; Yes and No. If a woman answered yes, then we assume that she has the ability to chart her menstrual cycle but if she answered no, we took it that she does not have the menstrual cycle charting ability.

## Results

### Socio-demographic characteristics of the respondents

Table 1 shows that most of the participants were aged between 24-29 years old when they got married (40%) and more than 36 years old (36%) at the time the survey was done. The age at menarche for more than half of the respondents was between 13 and 15 years. A good number of the respondents had a post graduate degree (45%) while about one fifth were unemployed (21%).

**Table 1:** Sociodemographic characteristics of the respondents

Descriptive variable	Frequency (n= 214)	Percentage (%)
**Age at menarche (years)**		
< 13	180	29
13-15	349	55
>15	100	16
**Age at marriage (years)**		
< 18	58	9
18-23	149	24
24-29	252	40
30-35	124	20
Above 35	48	7
**Age now (years)**		
< 25	60	10
25-30	163	26
31-36	177	28
> 36	625	36
**Religion**		
Christianity	531	85
Islam	84	13
Traditional Religion	10	2
**Geopolitical zones**		
FCT	17	3
North Central	70	11
Northwest	26	4
Northeast	12	2
South-South	231	36
Southeast	142	22
Southwest	143	22
**Highest Education level**		
Completed primary education	32	5
Completed secondary education	113	18
Completed post-secondary education	194	31
Completed post-graduate education	280	45
**Employment status**		
Unemployed	134	21
Employed	251	40
Self-employed	239	38

### Performance of the respondents to the individual awareness questions

In table 2, it is shown that some of the women were not aware of the fertile days in their cycle (28%) and quite a number of them were aware of the fertile cervical mucus type (47%). It is interesting to note that a low percentage of them were unaware that fertile days of the cycle can be learnt (17%). Overall, slightly above half of the respondents had a good awareness score (53%).

**Table 2:** Frequency distribution of the individual awareness questions

S/n	Statement	No Frequency (%)	Yes Frequency (%)
1	Aware of the fertile days in the menstrual cycle	180 (28)	460 (72)
2	Aware of ovulation pain	208 (32)	433 (68)
3	Aware of your cervical mucus secretions	210 (33)	431 (67)
4	Aware of the cervical mucus type which indicates fertility	300 (47)	341 (53)
5	Aware of ovulation detection through the urine	398 (62)	243(38)
6	Attempt to increase fertility awareness	245 (38)	396 (62)
7	Aware that doctors can educate them on fertility	171 (27)	469 (73)
8	Aware that they can discuss with their doctors about fertility	371 (58)	270 (42)
9	Awareness of intercourse timing to increase chances of pregnancy	162 (25)	479 (75)
10	Awareness that fertility days can be learnt	108 (17)	533 (83)
		**Poor Awareness**	**Good Awareness**
		303 (47)	338 (53)

### Level of fertility awareness

Table 3 shows a self-reported level of fertility awareness among the respondents, assuming that those who reported that they were aware of their fertility days of the menstrual cycle were actually so. There was a statistically significant association between the level of fertility awareness and age at menarche (X^2^ = 9.962, p = 0.007), geopolitical zone of residence (X^2^ = 17.301, p = 0.008), level of education (X^2^ = 64.843, p < 0.001), employment status (X^2^ = 9.319 p = 0.025) charting of the menstrual cycles (X^2^ = 66.392, p < 0.001), use of internet to increase awareness (X^2^ = 39.849, p < 0.001) and books (X^2^ = 58.855, p < 0.001). Table 4 shows the different medium the respondents used to increase their fertility awareness. There was a generally low interest in increasing their fertility awareness. None of the means were used by at least half of the respondents. For example, only 25% of them have checked the internet for fertility related enquiry.

**Table 3:** Association between the respondents' socio-demographics and the level of good fertility awareness

Descriptive variable	Total sample (N=641,(%)	Good Awareness (N=338, (%)	χ^2^ (P-value)
**Age at menarche (years)**			
< 13	180 (29)	98 (29)	9.962 (0.007)
13-15	349 (56)	198 (57)	
> 15	100 (16)	39 (12)	
**Religion**			
Christianity	531 (85)	288 (86)	5.007 (0.287)
Islam	84 (13)	42 (13)	
Traditional Religion	10 (2)	3 (1)	
**Geopolitical zones**			
FCT	17 (3)	15 (4)	
North Central	70 (11)	40 (12)	
Northwest	26 (4)	18 (5)	
Northeast	12 (2)	5 (2)	17.301 (0.008)
South-South	231 (36)	109 (32)	
Southeast	142 (22)	81 (24)	
Southwest	143 (22)	70 (21)	
**Highest Education level**			
Completed primary education	32 (5)	7 (2)	
Completed secondary education	113 (18)	30 (9)	64.843 (<0.001)
Completed post-secondary education	194 (31)	107 (32)	
Completed post-graduate education	280 (45)	186 (56)	
**Employment status**			
Unemployed	134 (21)	60 (18)	
Employed	251 (40)	150 (45)	9.319 (0.025)
Self-employed	239(38)	123 (37)	
**Menstrual Cycle charting ability**			
No	284 (45)	99 (30)	66.392 (<0.001)
Yes	347 (55)	235 (70)	
**Fertility awareness through the internet**			
No	480 (75)	218 (65)	39.849 (<0.001)
Yes	161 (25)	120 (35)	
**Fertility awareness through books**			
No	525 (82)	239 (71)	58.855 (<0.001)
Yes	116 (18)	99 (29)	
**Fertility awareness through doctor**			
No	509 (79)	237 (70)	36.537 (<0.001)
Yes	132 (21)	101 (30)	
**Fertility awareness through friend**			
No	552 (86)	280 (83)	5.849 (0.016)
Yes	89(14)	58 (17)	

**Table 4:** Means of increasing fertility awareness

S/n	Means	No Frequency (%)	Yes Frequency (%)
1	Books	525 (82)	116 (18)
2	Doctor	509 (79)	132 (21)
3	Friend	552 (86)	89 (14)
4	Internet	480 (75)	161 (25)
5	Natural Family planning teacher	611 (95)	30 (5)
6	Social media	640 (99)	1 (<1)
7	Nurse	638 (99)	3 (<1)
8	Fertility app	636 (99)	5 (1)
9	Pharmacist	638 (99)	3 (<1)

### Predictors of good fertility awareness level among the respondents

Table 5 contains the multiple logistic regression of the independent variables with fertility awareness level. Age at menarche, level of education, geopolitical zone of residence, menstrual cycle charting ability, fertility awareness through a doctor, a friend, the internet, and books were factors which predicted good fertility awareness. However, after adjusting for the independent variables, level of education and the ability to chart the menstrual cycle were predictors of good fertility awareness level. People with post graduate education had higher odds of having good fertility awareness level than those with secondary education (AOR=0.213, 95% CI 0.116-0.390, p < 0.001) while the odds of having good fertility awareness was less in those who did not chart their menstrual cycle (AOR=0.363, 95% CI 0.245-0.538, p < 0.001).

**Table 5:** Logistic Regression model of predictors of good fertility awareness level

Variables		COR (95% CI)	AOR (95% CI)
	Good Awareness (n=127)		
**Age Menarche (yrs.)**			
Less than 13	98 (29)	1.869 (1.137-3.074) *	1.757 (0.924-3.342)
13-15	198 (57)	2.051 (1.302-3.230) *	1.946 (1.069-3.541) *
More than 15	39 (12)	Reference	Reference
**Level of Education**			
Completed Primary	7 (2)	0.142 (0.059-0.339) **	0.318 (0.109-0.927) *
Completed secondary	30 (9)	0.183 (0.112-0.297) **	0.213 (0.116-0.390) **
Completed post-secondary	107 (32)	0.622 (0.427-0.906) *	0.778 (0.499- 1.211)
Completed post graduate	186 (56)	Reference	Reference
**Geopolitical zones**			
FCT	15 (4)	7.821 (1.725-35.455) *	4.647 (0.895-24.118)
North Central	40 (12)	1.390 (0.782-2.473)	0.982 (0.468-2.061)
Northwest	18 (5)	2.346 (.959-5.743)	0.807 (0.268-2.433)
Northeast	5 (2)	0.745 (0.226-2.457)	0.127 (0.025-0.649) *
South-South	109 (32)	0.932 (0.614-1.414)	0.913 (0.538-1.552)
Southeast	81 (24)	1.385 (0.868-2.209)	0.685 (0.379-1.237)
Southwest	70 (21)	Reference	Reference
**Employment Status**			
Unemployed	60 (18)	0.777 (0.509-1.187)	0.770 (0.443-1.340)
Employed	150 (45)	1.401 (0.979-2.003)	0.796 (0.504-1.257)
Self-employed	123 (37	Reference	Reference
**Menstrual Cycle charting ability**			
No	99 (30)	0.255 (0.183-0.355) **	0.363 (0.245-0.538) **
Yes	235 (70)	Reference	Reference
**Fertility awareness through a doctor**			
No	237 (70)	0.267 (0.172-0.415) **	0.776 (0.316-1.905)
Yes	101 (30)	Reference	Reference
**Fertility awareness through a friend**			
No	280 (83)	0.550 (0.345-0.878) *	1.863 (0.720-4.822)
Yes	58 (17)	Reference	Reference
**Fertility awareness through the internet**			
No	67 (53)	0.284 (0.191-0.423) **	1.008 (0.424-2.394)
Yes	60 (47)	Reference	Reference
**Fertility awareness through books**			
No	70 (55)	0.143 (0.083-0.247) **	0.458 (0.180-1.164)
Yes	57 (45)	Reference	Reference
**Number of sources of fertility information**		3.119 (2.460-3.9 54) **	2.514 (1.192-5.305) *

** p ≤ 0.01

* p ≤ 0.05

## Discussion

The aim of this survey was to measure the fertility awareness level among married Nigerian women of childbearing age and identify associating socio-demographic factors. The key findings were that about half of the women had a good fertility awareness level and that this was associated with the ability of the respondents to chart their menstrual cycle, their geopolitical zone of residence, age at menarche and level of education. Those who could chart their menstrual cycle had higher level of fertility awareness than those who did not chart theirs. Age at menarche between 13 -15 years predicted good fertility awareness among women than having age at menarche above 15 years. Those who had completed their post graduate studies had higher odds of good fertility awareness than those with only primary or secondary school education. Moreover, women residing in the Northeast region of Nigeria have lower odds of good fertility awareness than their counterpart in the Southwest region. In addition, an association was found between good fertility awareness level and employment status, use of internet and books to increase fertility awareness as well as through a doctor or a friend. However, of these, the highest predictors of good fertility awareness were menstrual cycle charting and level of education of the respondents.

Many of the respondents had married between 24-29 years of age. However, in a study by Olatoregun et al. (2014) in comparative analysis of fertility differentials in Ghana and Nigeria showed that a considerably higher proportion of the respondents got married between the ages 15-19 years^27^. This difference may have been because of the years apart between this study and the reference study. The latter was a retrospective study that made use of the 2008 Demographic and health surveys in Nigeria giving a 12-years gap between the periods of study. This current trend of a higher range of age at marriage may have been contributed by globalization. Many countries all over the world are experiencing a trend in increasing age at first child. In a study of fertility awareness and attitudes towards parenthood among Danish university college students by Sorensen et al (2016), the modal age range, when they wish to have their first child, was 25-29 years. Other past studies 15,28,29 in addition published similar findings of the age range of the first child. On the other hand, another survey observed an exceedingly higher age range (30-34 years old) among the respondents as, the modal desired age range for having the first child 30.

About half of the respondents had good fertility awareness. This result is higher than that obtained from a study by Hampton et al (2012) with a similar sample size, in Australia among women seeking fertility assistance (36.7%). In addition, women in this study over-estimated their knowledge 13. The respondents in our study may have also overstated their level of fertility awareness and there was no way of ascertaining the true knowledge level unlike in the Hampton et al study. It was found that the fertility awareness level has a bearing on women's attitudes towards fertility issues in general 31. Also, a moderate level of good fertility awareness can contribute to seeking less help related to fertility and more prone to fears about infertility among women of child bearing age^32^. It may also lead women not to appreciate the impact of increasing age on fertility issues. In fact, poor fertility awareness is a significant modifiable risk factor which when addressed can increase the chances of sub-fertile women of conceiving naturally thereby avoiding unnecessary assisted reproductive technologies (ART). It has also been shown that teaching fertility awareness can be effective, safe, and less costly, making it ideal for couples who cannot access ART or choose not to resort to it because of cultural or religious convictions 13.

After controlling for co-founders in the logistic regression, the ability of women to chart their menstrual cycle, age at menarche, educational level, geopolitical zones of residence and increasing number of sources of fertility information emerged as predictors of good fertility awareness. Consistent with this conclusion is a comprehensive literature review by The Institute for Reproductive health, USA which indicated a global lack of fertility awareness attributable to the lack of charting ability 33. It has also been demonstrated that awareness of menstruation does not automatically lead to awareness of the fertile period in the cycle 18. One good way of increasing the fertility awareness of the women is through receiving effective training by experts to chart accurately the menstrual cycle. Women in this study who had completed their post graduate education had higher fertility awareness than their counterparts with primary or secondary education. A similar finding was made by Swift et al which showed a linear relationship between the level of education and fertility awareness^34^. People residing in the North-East geopolitical zones were disadvantaged; information which is important in planning intervention programs. Women from the North-East region have also suffered from insurgences, with the majority of the inhabitants internally displaced which leads to poverty and health illiteracy^35^; factors which most likely will influence fertility awareness.

## Conclusion

About half of the women had a good fertility awareness level which was associated with the ability of the respondents to chart their menstrual cycle, their geopolitical zone of residence, age at menarche and level of education. Those who could chart their menstrual cycle had higher level of fertility awareness than those who did not chart theirs. Age at menarche between 13 -15 years predicted good fertility awareness among women than age at menarche above 15 years. Those who had completed their post graduate studies had higher odds of good fertility awareness than those with only primary or secondary school education. Moreover, women residing in the Northeast region of Nigeria have lower odds of good fertility awareness than their counterpart in the Southwest region. In addition, an association was found between good fertility awareness level and employment status, use of internet and books to increase fertility awareness as well as through a doctor or a friend. However, of these, the highest predictors of good fertility awareness among women of childbearing age in Nigeria were menstrual cycle charting and level of education of the respondents.

### Implications of the findings for community and public health Pharmacists

Pharmacists in the community as well as in public health have been involved in family planning outreach. They can also be empowered through Fertility Education Programs to provide fertility awareness advice to women especially at basic level. This is crucial because they are usually the first point of call for women who desire to have children and are seeking supplements and vitamins to enable them to achieve pregnancy. Such encounter could provide an opportunity for offering counselling services with respect to fertility awareness.
